# Prevalence of anemia in patients with chronic kidney disease in Japan: A nationwide, cross-sectional cohort study using data from the Japan Chronic Kidney Disease Database (J-CKD-DB)

**DOI:** 10.1371/journal.pone.0236132

**Published:** 2020-07-20

**Authors:** Tadashi Sofue, Naoki Nakagawa, Eiichiro Kanda, Hajime Nagasu, Kunihiro Matsushita, Masaomi Nangaku, Shoichi Maruyama, Takashi Wada, Yoshio Terada, Kunihiro Yamagata, Ichiei Narita, Motoko Yanagita, Hitoshi Sugiyama, Takashi Shigematsu, Takafumi Ito, Kouichi Tamura, Yoshitaka Isaka, Hirokazu Okada, Kazuhiko Tsuruya, Hitoshi Yokoyama, Naoki Nakashima, Hiromi Kataoka, Kazuhiko Ohe, Mihoko Okada, Naoki Kashihara

**Affiliations:** 1 Division of Nephrology and Dialysis, Department of Cardiorenal and Cerebrovascular Medicine, Kagawa University, Kagawa, Japan; 2 Division of Cardiology, Nephrology, Respiratory and Neurology, Department of Internal Medicine, Asahikawa Medical University, Asahikawa, Japan; 3 Medical Science, Kawasaki Medical School, Kurashiki, Japan; 4 Department of Nephrology and Hypertension, Kawasaki Medical School, Kurashiki, Japan; 5 Department of Epidemiology, Johns Hopkins Bloomberg School of Public Health, Baltimore, Maryland, United States of America; 6 Division of Nephrology and Endocrinology, University of Tokyo Graduate School of Medicine, Tokyo, Japan; 7 Division of Nephrology, Nagoya University Graduate School of Medicine, Nagoya, Japan; 8 Division of Nephrology, Department of Nephrology and Laboratory Medicine, Kanazawa University, Kanazawa, Japan; 9 Department of Endocrinology, Metabolism and Nephrology, Kochi Medical School, Kochi University, Kochi, Japan; 10 Department of Nephrology, Faculty of Medicine, University of Tsukuba, Tsukuba, Japan; 11 Division of Clinical Nephrology and Rheumatology, Niigata University Graduate School of Medical and Dental Sciences, Niigata, Japan; 12 Department of Nephrology, Graduate School of Medicine, Kyoto University, Kyoto, Japan; 13 Department of Human Resource Development of Dialysis Therapy for Kidney Disease, Okayama University Graduate School of Medicine, Dentistry and Pharmaceutical Sciences, Okayama, Japan; 14 Division of Nephrology, Department of Internal Medicine, Wakayama Medical University, Wakayama, Japan; 15 Division of Nephrology, Faculty of Medicine, Shimane University, Izumo, Japan; 16 Department of Medical Science and Cardiorenal Medicine, Yokohama City University Graduate School of Medicine, Yokohama, Japan; 17 Department of Nephrology, Osaka University Graduate School of Medicine, Suita, Japan; 18 Department of Nephrology, Faculty of Medicine, Saitama Medical University, Saitama, Japan; 19 Department of Integrated Therapy for Chronic Kidney Disease, Kyushu University, Fukuoka, Japan; 20 Department of Nephrology, Nara Medical University, Kashihara, Japan; 21 Department of Nephrology, Kanazawa Medical University School of Medicine, Ishikawa, Japan; 22 Department of Advanced Information Technology, Kyushu University, Fukuoka, Japan; 23 Faculty of Health Science and Technology, Kawasaki University of Medical Welfare, Kurashiki, Japan; 24 Department of Healthcare Information Management, The University of Tokyo Hospital, Tokyo, Japan; 25 Institute of Health Data Infrastructure for All, Tokyo, Japan; Tokushima University Graduate school, JAPAN

## Abstract

**Background:**

The Japan Chronic Kidney Disease Database (J-CKD-DB) is a nationwide clinical database of patients with chronic kidney disease (CKD) based on electronic health records. The objective of this study was to assess the prevalence of anemia and the utilization rate of erythropoiesis-stimulating agents (ESAs) in Japanese patients with CKD.

**Methods:**

In total, 31,082 adult outpatients with estimated glomerular filtration rates of 5–60 ml/min/1.73 m^2^ in seven university hospitals were included this analysis. The proportions of patients with CKD stages G3b, G4, and G5 were 23.5%, 7.6%, and 3.1%, respectively.

**Results:**

The mean (standard deviation) hemoglobin level of male patients was 13.6 (1.9) g/dl, which was significantly higher than the mean hemoglobin level of female patients (12.4 (1.6) g/dl). The mean (standard deviation) hemoglobin levels were 11.4 (2.1) g/dl in patients with CKD stage G4 and 11.2 (1.8) g/dl in patients with CKD stage G5. The prevalences of anemia were 40.1% in patients with CKD stage G4 and 60.3% in patients with CKD stage G5. Logistic regression analysis showed that diagnoses of CKD stage G3b (adjusted odds ratio [95% confidence interval]: 2.32 [2.09–2.58]), G4 (5.50 [4.80–6.31]), and G5 (9.75 [8.13–11.7]) were associated with increased prevalence of anemia. The utilization rates of ESAs were 7.9% in patients with CKD stage G4 and 22.4% in patients with CKD stage G5.

**Conclusions:**

We determined the prevalence of anemia and utilization rate of ESAs in Japanese patients with CKD using data from a nationwide cohort study.

## Introduction

A reduction in estimated glomerular filtration rate (eGFR) <60 ml/min/1.73 m^2^ is regarded as a sign of chronic kidney disease (CKD), as are structural or functional renal abnormalities [1]. Approximately 13% of the Japanese adult population is estimated to have CKD [2]. Cross-sectional estimates of the prevalence of CKD in the United States range from 1.5% to 15.6% [3]. CKD is categorized on the basis of eGFR and the degree of proteinuria [4], and is reportedly a major risk factor for cardiovascular disease [5]. Moreover, the socioeconomic impact of CKD is an important global problem [6].

An important function of the kidneys is the production of erythropoietin, a signaling molecule that stimulates red blood cell production, in response to reduced levels of oxygen in the blood. The level of hemoglobin is lower in patients with CKD than in the normal population [7]. Other possible causes of anemia in patients with CKD include iron deficiency, inflammation, and uremic toxin accumulation [7, 8]. Anemia is a predictor of CKD progression, cardiovascular disease mortality [9, 10], and cardio-renal-anemia syndrome [11, 12] in patients with CKD. Treatment of anemia by erythropoiesis-stimulating agents (ESAs) is expected to reduce mortality and cardiovascular disease risk in patients with CKD [13].

The prevalence of anemia and utilization rate of ESAs in patients with CKD have been reported [14–17]. In general, large-scale studies involve potential input errors and extensive burdens on physicians. However, the use of a data extraction and registration system may avoid the risk of input error. In the past several years, analyses of the real-world condition in patients with CKD were performed based on electronic health records [18–21], and the prevalence of anemia among these patients was reported [22, 23]. Despite these previous reports, similar studies using electronic health records in patients with CKD have not yet been conducted in Japan.

The Japan Chronic Kidney Disease Database (J-CKD-DB) is a large-scale, nationwide comprehensive clinical database of patients with CKD based on electronic health records data from participating university hospitals, constructed by the Japanese Society of Nephrology and the Japan Association for Medical Informatics [24]. Using a standardized method for information exchange (Standardized Structured Medical Information eXchange; SS-MIX2) [25], the J-CKD-DB efficiently compiles clinical data of patients with CKD across hospitals, regardless of differences in electronic health records systems. Instead of manually input data regarding selected patients undergoing specialist care, the CKD-DB cohort includes automatically extracted data regarding all patients with CKD in university hospitals in Japan. Therefore, the J-CKD-DB enables investigations of real-world CKD treatment practices in Japan through both cross-sectional and prospective studies.

The aim of this study was to assess the real-world prevalence of anemia according to different international criteria for renal anemia, as well as the utilization rate of ESAs, in Japanese patients with CKD. This study used automatically extracted data from a nationwide, large-scale cohort study in Japan.

## Materials and methods

### Study setting and participants

The J-CKD-DB is a multicenter, automatically extracted comprehensive database of patients with CKD from 21 university hospitals in Japan (UMIN trial number, UMIN000026272) [24]. The inclusion criteria for the J-CKD-DB are as follows: 1) age ≥18 years; 2) proteinuria ≥1+ (dipstick test) and/or eGFR <60 ml/min/1.73 m^2^, where eGFR is calculated using the Japanese eGFR equation: eGFR (ml/min/1.73 m^2^) = 194 × serum creatinine value ^-1.094^ × age—^0.287^ (× 0.739 [for women]) [26]. All data elements are extracted in a semiautomatic manner via the SS-MIX2 format and stored in the Multipurpose Clinical Data Registry System [24, 27]. Patients undergoing renal replacement therapy (i.e., hemodialysis, peritoneal dialysis, and kidney transplantation) are manually omitted.

In this study, we performed an observational cross-sectional investigation as an initial analysis of data from the J-CKD-DB. To exclude most patients with acute kidney injury, we omitted inpatients from this analysis. We identified 39,121 adult outpatients with at least one measurement of serum creatinine in seven university hospitals from January 1, 2014 to December 31, 2014. We included 35,508 patients with CKD based on outpatient laboratory data that indicated eGFR <60 ml/min/1.73 m^2^ (using the lowest value of eGFR) [28, 29]. In the initial analysis, patients with eGFR <5 ml/min/1.73 m^2^ were excluded to eliminate the possibility of including patients with end-stage renal disease, because we could not distinguish patients receiving hemodialysis or peritoneal dialysis, or patients who had undergone kidney transplantation, at the time of the initial analysis. Hemoglobin levels were assessed in 31,082 (88%) of 35,508 patients. Finally, 31,082 eligible outpatients were included in this observational cross-sectional study.

### Data collection

CKD G categories were defined as follows: stage G3a, eGFR of ≥45 and <60 ml/min/1.73 m^2^; stage G3b, eGFR of ≥30 and <45 ml/min/1.73 m^2^; stage 4, eGFR of ≥15 and <30 ml/min/1.73 m^2^; and stage 5, eGFR <15 ml/min/1.73 m^2^ [1]. eGFR5 categories were divided into 11 groups with eGFR ranges of 5 ml/min/1.73 m^2^ each, ranging from eGFR of ≥55 and <60 ml/min/1.73 m^2^ to eGFR of ≥5 and <10 ml/min/1.73 m^2^. Dipstick proteinuria results were classified into three categories: KDIGO A1 category of negative proteinuria (-); A2 category of trace proteinuria (±); and A3 category of ≥1+ [30].

Data obtained from the SSMIX-2 system for this analysis were age, sex, eGFR, dipstick proteinuria, hemoglobin, serum albumin, total cholesterol, sodium, potassium, chloride, calcium, phosphate, C-reactive protein (CRP), and use of ESAs. The lowest value of eGFR during the study period was selected as the eGFR value for analysis. Data without corresponding eGFR values were extracted for 1 week before or 1 week after an eGFR value was recorded (22). The difference between sodium and chloride concentrations (Na–Cl (mEq/L)) was calculated. The mean hemoglobin level was analyzed based on G category, eGFR5 category, age, and sex strata. The range for optimal hemoglobin level was defined as ≥11.0 g/dl in patients without ESAs, whereas it was ≥11.0 and <13.0 g/dl in patients undergoing ESA treatment, based on Japanese clinical practice guidelines (CPGs) during the study period [31, 32]. These CPGs also recommended initiation of treatment with ESAs for patients with CKD whose hemoglobin levels were <11.0 g/dl.

Anemia was defined by the following four sets of criteria: JSDT1 Criteria, Japanese Society for Dialysis Therapy (JSDT) criteria for renal anemia [31]—hemoglobin level ≤13.5 g/dl for men aged 19–59 years, ≤12.0 g/dl for men aged 60–69 years, ≤11.0 g/dl for men aged ≥70 years, ≤11.5 g/dl for women aged 19–59 years, and ≤10.5 g/dl for women aged ≥60 years; KDOQI Criteria, Kidney Disease Outcomes Quality Initiative (KDOQI) CPG [8]: hemoglobin level ≤13.5 g/dl for men aged 19–59 years and ≤12.0 g/dl for women; EBPG Criteria, European Best Practice Guidelines (EBPG) 2004 anemia guideline [33]: hemoglobin level ≤13.5 g/dl for men aged <69 years, ≤12.5 g/dl for men aged ≥70 years, and ≤11.5 g/dl for women; and JSDT2 Criteria, based on the requirement for or use of ESAs: hemoglobin level ≤11.0 g/dl (criteria for initiation of ESA according to the JSDT [31]) or ongoing treatment with ESAs.

### Ethics statement

The J-CKD-DB study was comprehensively approved by the ethics committee of Kawasaki Medical School and Japanese Society of Nephrology (JSN-28) and individually approved by local committee of participating university hospitals (Kagawa University Hospital, Asahikawa Medical University Hospital, University of Tokyo Graduate School of Medicine, Nagoya University Hospital, Kanazawa University Hospital, Kochi University Hospital, University of Tsukuba Hospital, Niigata University Hospital, Kyoto University Hospital, Okayama University Hospital, Wakayama Medical University Hospital, Shimane University Hospital, Yokohama City University Hospital, Osaka University Hospital, Saitama Medical University Hospital, Nara Medical University Hospital, Kanazawa Medical University Hospital, and Kyushu University Hospital). The study was conducted in accordance with the ethical principles of the World Medical Association Declaration of Helsinki. Informed consent was obtained in the form of opt-out on the website of each participating university hospital. Patients who declined to participate in the J-CKD-DB were not registered.

### Statistical analysis

Values are presented as medians with interquartile intervals, means with standard deviations, or counts with percentages, as appropriate. Distributions of variables were evaluated by histogram, quantile-quantile plot, and the Kolmogorov–Smirnov test. Clinical variables were compared between groups using the χ^2^ test for categorical variables and Student’s t-test, one-way analysis of variance, two-way analysis of variance, or the Mann–Whitney U test for continuous variables. Tukey's multiple comparison test was used for post hoc corrections for multiple comparisons. All data were statistically analyzed using IBM SPSS Advanced Statistics, version 27.0 (IBM Corp., Armonk, NY, USA), and p<0.05 was considered to indicate significant differences.

To identify the independent association of G category with the prevalence of anemia, multivariable logistic regression models were used. Model 1 (including age, A category, albumin level, and CRP as covariates) was used for JSDT1 Criteria, KDOQI Criteria, and EBPG Criteria; model 2 (including age, sex, A category, albumin level, and CRP as covariates) was used for JSDT2 Criteria. The adjusted odds ratios and corresponding two-sided 95% confidence intervals of the predictors are shown. To identify the effects of factors on the prevalence of anemia (JSDT2 Criteria), a logistic regression analysis model (model 3) was used; this model included age ≥65 years, female sex, G category, albumin level ≤3.5 g/dl, difference between sodium and chloride concentrations (Na–Cl) ≤30 mEq/L, and CRP level ≥0.3 mg/dl as covariates.

## Results

### Baseline characteristics of enrolled outpatients

Baseline characteristics of enrolled outpatients are shown in Table 1. The median age was 72 [interquartile interval, 64–79] years, 54.5% were men, and median eGFR was 50.0 [interquartile interval, 40.9–55.6] ml/min/1.73 m^2^. The numbers of patients with CKD stages G3a, G3b, G4, and G5 were 23,333 (65.7%), 8,357 (23.5%), 2,710 (7.6%), and 1,108 (3.1%), respectively. Although there was limited proteinuria data (43% of all patients [i.e., 15,442]), the numbers of patients with CKD stages A1, A2, and A3 were 9,357 (60.6%), 2,295 (14.9%), and 3,790 (24.5%), respectively. The mean hemoglobin level (standard deviation) of all patients was 13.0 (1.9) g/dl; the mean hemoglobin level (standard deviation) of male patients was 13.6 (1.9) g/dl, which was significantly higher than the mean hemoglobin level of female patients (12.4 [1.6] g/dl). Stratification of patients according to eGFR category, age, and sex strata is shown in S1 Table.

**Table 1:** General characteristics of Japanese outpatients with chronic kidney disease.

*n*	35,508
Age (years)	72.0 [64.0–79.0]
Age category	
18–45 years	1,173 (3.3%)
45–64 years	7,966 (22.4%)
65–74 years	11,628 (32.7%)
75–84 years	11,309 (31.8%)
≥85 years	3,432 (9.7%)
Sex: male	19,360 (54.5%)
eGFR (ml/min/1.73 m^2^)	50.0 [40.9–55.6]
G category	
G3a	23,333 (65.7%)
G3b	8,357 (23.5%)
G4	2,710 (7.6%)
G5	1,108 (3.1%)
Dipstick proteinuria	Overall: 15,442
(-)	9,357 (60.6%)
(±)	2,295 (14.9%)
(1+)	1,849 (12.0%)
(2+)	1,277 (8.3%)
(3+)	598 (3.9%)
(4+)	66 (0.4%)
Hemoglobin (g/dl)	13.02 (1.88)
Serum albumin (g/dl)	4.04 (0.47)
Serum uric acid (mg/dl)	6.05 (1.49)
Serum total cholesterol (mg/dl)	187.9 (38.3)
Serum sodium	141.0 (2.84)
Serum potassium	4.41 (0.54)
Serum chloride	104.6 (3.34)
Serum calcium	9.08 (0.54)
Serum phosphate	3.52 (0.94)
Serum C-reactive protein	0.10 [0.04–0.28]

Continuous variables are described as median [interquartile interval] or mean (standard deviation). Categorical variables are described as n (%).

Abbreviation: eGFR, estimated glomerular filtration rate.

### Association between hemoglobin level and eGFR

**Fig 1:**
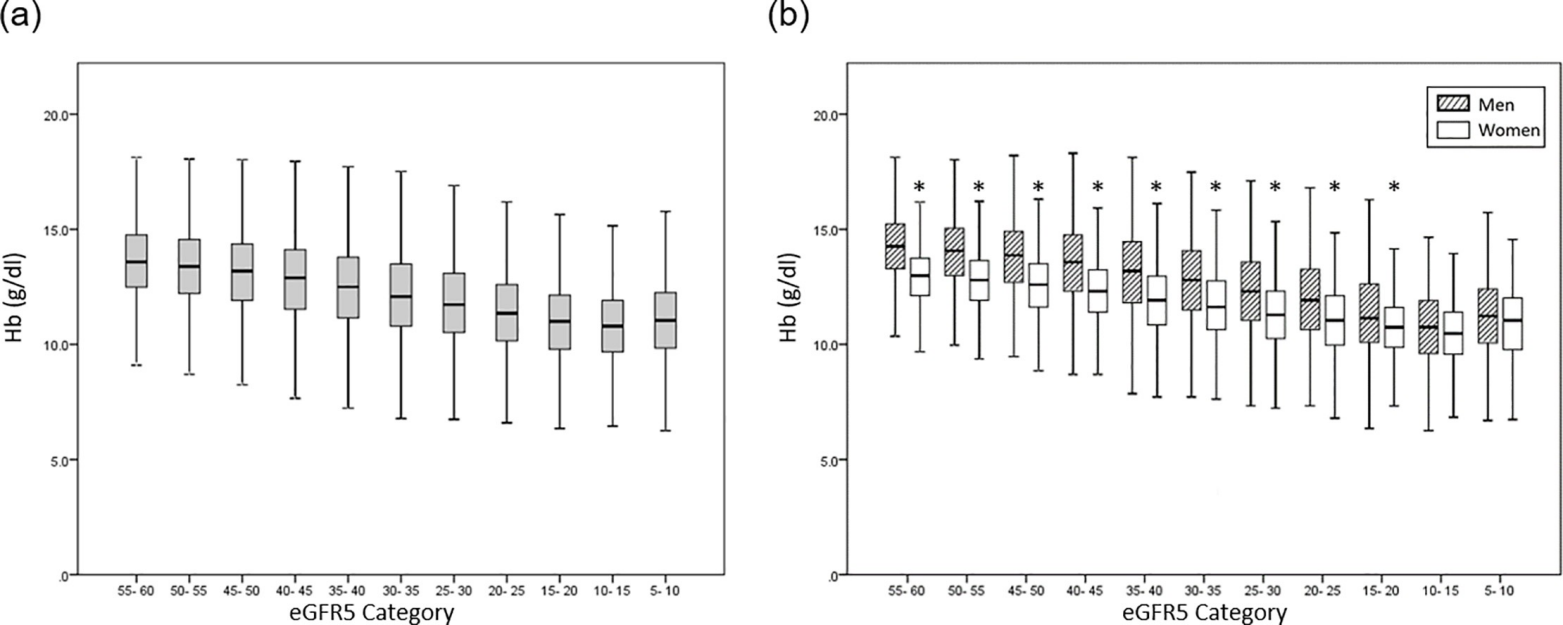
Mean hemoglobin level according to eGFR5 category. (a) All participants; (b) Men and women shown separately. Hb, hemoglobin; *, p<0.05.

**Fig 2:**
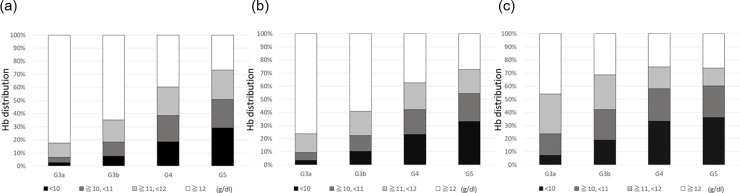
Hemoglobin distribution according to G category. (a) All participants; (b) Men; and (c) Women. Hb, hemoglobin.

Mean hemoglobin levels according to eGFR5 category and sex strata are shown in Fig 1. The mean hemoglobin level was reduced in accordance with the progression of eGFR5 category in both male and female patients. However, the mean hemoglobin level was higher in patients with an eGFR5 category of ≥10 and <15 ml/min/1.73 m^2^ than in patients with an eGFR5 category of ≥5 and <10 ml/min/1.73 m^2^. The mean hemoglobin level was higher in male patients than in female patients, except in patients with an eGFR5 category of ≥10 and <15 ml/min/1.73 m^2^, and in patients with an eGFR5 category of ≥5 and <10 ml/min/1.73 m^2^. Hemoglobin distribution according to G category and sex strata is shown in Fig 2. The proportion of patients with low hemoglobin level increased in accordance with the progression of G category in both male and female patients.

The mean hemoglobin levels according to G category, age, and sex strata are shown in S2 Table. The mean hemoglobin level gradually decreased in accordance with the progression of G category; moreover, the mean hemoglobin level was significantly lower in female patients than in male patients in all G categories. The mean hemoglobin levels were significantly lower in patients aged 75–84 years and patients aged ≥85 years than in patients aged 45–64 years in each G category. The mean hemoglobin levels according to A category strata are shown in S3 Table. In each G category, there was no significant difference in mean hemoglobin level based on the progression of A category.

Since there were few patients with severe proteinuria (urinary total protein ≥3+ and albumin <3 g/dl) and high inflammatory levels (CRP ≥5 mg/dl) in this analysis, the mean (standard deviation) hemoglobin levels did not significantly change when those patients were excluded from analysis (S4 Table).

### Prevalence of anemia

The prevalences of anemia according to G category, age, and sex strata based on four different sets of criteria for anemia are shown in Table 2. For each set of criteria, the prevalence of anemia increased in accordance with the progression of G category. The prevalences of anemia according to KDOQI Criteria and EBPG Criteria were similar to those according to JSDT1 criteria. Using JSDT1 Criteria and KDOQI Criteria, the prevalences of anemia were higher in male patients than in female patients because of the different thresholds used for men and women in these sets of criteria. Using JSDT2 Criteria (requirement for or use of ESAs), the prevalence of anemia was higher in female patients than in male patients. According to KDOQI, EBPG, and JSDT2 Criteria, the prevalences of anemia were significantly higher in patients aged 75–84 years and in patients aged ≥85 years, compared with patients aged 45–64 years, in each G category. According to JSDT1 Criteria, the prevalences of anemia were higher in patients aged 18–44 than in patients aged 45–64 in the G3a, G3b, and G4 categories.

**Table 2:** Prevalences of anemia according to G category, age, and sex strata.

**JSDT1 Criteria**									
		18–44 Y	45–64 Y	65–74 Y	75–84 Y	≥85 Y	Men	Women	Total
G3a	%	20.7^†^	11.1	4.5^†^	6.5^†^	12.5	8.4	7.2^‡^	7.8
G3b	%	40.7^†^*	24.3*	12.9^†^*	16.1^†^*	22.5*	18.4*	17.6*	18.1*
G4	%	57.1^†^*	44.3*	32.8^†^*	37.6^†^*	46.1*	41.4*	38.5*	40.1*
G5	%	74.7*	65.3*	53.5^†^*	52.5^†^*	73.9*	67.8*	49.3*^‡^	60.3*
**KDOQI Criteria**									
		18–44 Y	45–64 Y	65–74 Y	75–84 Y	≥85 Y	Men	Women	Total
G3a	%	18.8^†^	11.1	13.4^†^	23.3^†^	38.9^†^	10.6	26.3^‡^	17.7
G3b	%	38.3^†^*	24.5*	28.8^†^*	39.1^†^*	52.0^†^*	25.6*	47.7^‡^*	35.4*
G4	%	47.1*	49.4*	54.6*	65.2^†^*	76.5^†^*	50.3*	72.7^‡^*	60.7*
G5	%	64.2*	63.4*	78.3^†^*	80.8^†^*	87.0^†^*	69.9*	78.9^‡^*	73.5*
**EBPG Criteria**									
		18–44 Y	45–64 Y	65–74 Y	75–84 Y	≥85 Y	Men	Women	Total
G3a	%	20.7^†^	16.6	17.4	21.7^†^	37.4^†^	23.1	16.1^‡^	20.0
G3b	%	40.7*	34.3*	32.1*	38.5^†^*	51.1^†^*	39.6*	35.3^‡^*	37.7*
G4	%	57.1*	58.9*	60.0*	65.1^†^*	76.9^†^*	67.6*	60.6^‡^*	64.3*
G5	%	74.7*	76.4*	82.6*	85.0^†^*	89.9^†^*	89.6*	68.4^‡^*	81.0*
**JSDT2 Criteria (Hb ≤11.0 g/dl or use of ESAs)**									
		18–44 Y	45–64 Y	65–74 Y	75–84 Y	≥85 Y	Men	Women	Total
G3a	%	9.3^†^	4.0	4.3	7.7^†^	13.7^†^	4.0	8.5^‡^	6.1
G3b	%	24.7^†^*	11.9*	12.5*	17.8^†^*	23.6^†^*	12.2*	21.4^‡^*	16.3*
G4	%	34.4*	26.4*	30.5*	41.9^†^*	48.8^†^*	30.1*	44.9^‡^*	37.1*
G5	%	46.5*	43.4*	52.8^†^*	61.6^†^*	71.4^†^*	49.5*	57.8^‡^*	52.9*

Prevalences of anemia are expressed as % of each population and were analyzed by the chi‐squared test.

*:p<0.05 vs. G3a

†:p<0.05 vs. 45–64 Y

‡:P<0.05 vs. Men.

Abbreviations: JSDT, Japanese Society for Dialysis Therapy; KDOQI, Kidney Disease Outcomes Quality Initiative; EBPG, European Best Practice Guidelines; ESA, erythropoiesis-stimulating agent.

Adjusted odds ratios and 95% confidence intervals for increased prevalence of anemia according to G category strata are shown in Tables 3 and 4. Multiple regression analysis using models 1 and 2 showed that the prevalence of anemia gradually increased in accordance with the progression of G category in each set of criteria, independent of age, A category, albumin level, CRP, and sex (notably, sex was only included for JSDT2 Criteria) (Table 3). Using JSDT1 Criteria, the adjusted odds ratio and 95% confidence interval for increased prevalence of anemia were 2.32 [2.09–2.58] in patients with CKD stage G3b, 5.50 [4.80–6.31] in patients with CKD stage G4, and 9.75 [8.13–11.7] in patients with CKD stage G5. Logistic regression analysis using model 3 (Table 4) showed that age ≥65 years, female sex, albumin level ≤3.5 g/dl, difference between sodium and chloride concentrations (Na–Cl) ≤30 mEq/L, and CRP level ≥0.3 mg/dl were also independent risk factors for increased prevalence of anemia (JSDT2 Criteria).

**Table 3:** Adjusted odds ratios and 95% confidence intervals for increased prevalence of anemia based on G category strata.

		Anemia (JSDT1 Criteria) *	Anemia (KDOQI Criteria)*	Anemia (EBPG Criteria)*	Anemia (JSDT2 Criteria)^†^
G3a	Reference	1	1	1	1
G3b	AOR [95% CI]	2.32 [2.09–2.58]	2.02 [1.86–2.20]	2.03 [1.87–2.21]	2.26 [2.03–2.52]
G4	AOR [95% CI]	5.50 [4.80–6.31]	4.89 [4.31–5.55]	4.88 [4.31–5.54]	5.09 [4.45–5.82]
G5	AOR [95% CI]	9.75 [8.13–11.7]	9.30 [7.67–11.3]	11.2 [9.15–13.8]	10.6 [8.85–12.7]

Model 1*: age, CKD A category, Alb, and CRP as covariates.

Model 2^†^: age, sex, CKD A category, Alb, and CRP as covariates.

AOR and 95% CI were analyzed by logistic regression analysis using each of the above factors as covariates.

Abbreviations: Alb, albumin; AOR, adjusted odds ratio; CI, confidence interval, CKD, chronic kidney disease; CRP, C-reactive protein; JSDT, Japanese Society for Dialysis Therapy; KDOQI, Kidney Disease Outcomes Quality Initiative; EBPG, European Best Practice Guidelines.

**Table 4:** Adjusted odds ratios and 95% confidence intervals for increased prevalence of anemia using JSDT2 criteria.

		AOR [95% CI]
Age (years)	≥65	1.65 [1.46–1.85]
Sex	Female	2.33 [2.11–2.56]
G category	G3a	1
	G3b	2.44 [2.18–2.72]
	G4	6.65 [5.82–7.61]
	G5	12.8 [10.6–15.3]
Albumin	≤3.5	5.36 [4.74–6.05]
Na-Cl	≤30	3.41 [2.44–4.76]
CRP	≥0.3	1.59 [1.42–1.78]

Model 3: age ≥65, female sex, G category, albumin level ≤3.5 g/dl, difference between sodium and chloride concentrations (Na–Cl) ≤30 mEq/L, and CRP level ≥0.3 mg/dl as covariates.

AOR and 95% CI were analyzed by logistic regression analysis using each of the above factors as covariates.

Abbreviations: AOR, adjusted odds ratio; CI, confidence interval, CKD, chronic kidney disease; CRP, C-reactive protein; JSDT, Japanese Society for Dialysis Therapy.

### Rate of hemoglobin level within optimal range and utilization rate of ESAs

The rates of hemoglobin levels within optimal ranges are shown in Table 5; these were 54.6% in patients with CKD stage G4, 44.8% in patients with CKD stage G5, and 51.7% in patients with CKD stage G4+5. The rates of hemoglobin levels within optimal ranges in patients with CKD stage G4+5 were 46.9% in patients aged 75–84 years, 37.7% in patients aged ≥85 years, and 44.4% in female patients.

**Table 5:** Rates of hemoglobin levels within optimal ranges (≥11.0 g/dl in Patients without ESA; ≥11.0 g/dl and <13 g/dl in patients with ESA) according to G category, age, and sex strata.

		18–44 Y	45–64 Y	65–74 Y	75–84 Y	≥85 Y	Men	Women	Subtotal
G3a	%	82.5^†^	83.9	83.5	78.6^†^	71.3^†^	84.3	77.6^‡^	81.2
G3b	%	69.5*	78.6*	76.3*	69.3^†^*	60.5^†^*	76.5*	65.6*^‡^	71.6*
G4	%	62.6*	66.7*	60.0*	50.4^†^*	40.5^†^*	62.3*	45.8*^‡^	54.6*
G5	%	52.5*	54.5*	46.6*	35.1^†^*	19.5^†^*	47.8*	40.5*^‡^	44.8*
G4+G5	%	58.2*	61.8*	55.7^†^*	46.9^†^*	37.7^†^*	57.8*	44.4*^‡^	51.7*

Rates are expressed as % of each population.

*:p<0.05 vs. G3a

†:p<0.05 vs. 45–64 Y

‡:p<0.05 vs. Men.

Abbreviations: ESA, erythropoiesis-stimulating agent; Hb, hemoglobin.

The utilization rates of ESAs according to G category and age strata are shown in Table 6. Although the ESA utilization rate increased in accordance with the progression of G category, the ESA utilization rates were low: 7.9% of patients with CKD stage G4 and 22.4% of patients with CKD stage G5. The rate of ESA non-use in patients with CKD stage G4+5 who had hemoglobin level ≤11.0 g/dl was 77.5% (S5 Table). Moreover, the rates of ESA non-use were higher in patients aged 75–84 years and ≥85 years.

**Table 6:** Utilization rates of ESAs according to G category and age strata.

		18–44 Y	45–64 Y	65–74 Y	75–84 Y	≥85 Y	Subtotal
G3a	%	0.1	0.1	0.0	0.0	0.1	0.0
G3b	%	2.2*	0.8*	0.8*	0.6*	0.7*	0.7*
G4	%	10.7*	7.2*	5.8*	9.0*	8.5*	7.9*
G5	%	23.8^†^*	16.3*	23.9^†^*	29.0^†^*	16.9*	22.4*
G4+G5	%	16.4^†^*	10.8*	11.6*	13.7*	9.6*	12.1*

Utilization rates are expressed as % of each population.

*:p<0.05 vs. G3a

†:p<0.05 vs. 45–64 Y.

Abbreviation: ESA, erythropoiesis-stimulating agent.

The mean hemoglobin level in patients with CKD stage G4 undergoing treatment with ESAs was 10.1 g/dl, which was significantly lower than the mean hemoglobin level in patients with CKD stage G4 without ESAs (11.7 g/dl). The mean hemoglobin level in patients with CKD stage G5 undergoing treatment with ESAs was 10.1 g/dl, which was significantly lower than the mean hemoglobin level in patients with CKD stage G5 without ESAs (11.1 g/dl).

## Discussion

In this study, we demonstrated the prevalence of anemia according to different international criteria for renal anemia, as well as the utilization rate of ESAs, in 31,082 Japanese patients with CKD by using automatically extracted data from a nationwide, large-scale cohort study in Japan. We previously investigated the prevalence, related factors, and management of anemia in Japanese patients with CKD stages 3–5 (n = 2,930) by using baseline data obtained from a prospective cohort study (Chronic Kidney Disease Japan Cohort) [14]. In contrast to previous studies in Japan, the J-CKD-DB study allows large-scale analyses directly linked to the electronic medical record using the SS-MIX2 standard; importantly, it is a precursor to a large-scale analysis of renal anemia in Japanese patients with CKD.

The present study demonstrated that the difference in hemoglobin level between male and female patients decreased in accordance with the progression of G category. In contrast, there remained a difference in hemoglobin level between sexes in patients with CKD stages G4 and 5. Notably, the degree of proteinuria was not associated with hemoglobin levels in our study. The present study also demonstrated that the prevalence of anemia in Japanese patients with CKD was similar to the prevalence of their counterparts in a cohort study in the United States [22, 34], although the prevalence of anemia differed on the basis of the set of criteria used. Multiple regression analysis revealed that the progression of G category was a significant risk factor for anemia; other risk factors included female sex, low serum albumin level, narrower difference between sodium and chloride concentrations, and high serum CRP level. This narrower difference between sodium and chloride concentrations may be associated with the presence of metabolic acidosis [35]. Our data suggested that poor nutritional status, metabolic acidosis, and inflammation were also risk factors for anemia.

Severe proteinuria and high inflammatory levels are also considered risk factors for acute kidney injury and anemia. The purpose of this analysis was to determine the real-world status of outpatients with CKD. To exclude patients receiving dialysis, we excluded patients receiving dialysis and post-transplant patients, as well as patients with eGFR ≤5 ml/min/1.73 m^2^. We cannot exclude the possibility that patients with acute kidney injury due to severe proteinuria and high inflammatory response may have been included among “outpatients with CKD”; however, to ensure diversity among outpatients with CKD, our analysis included patients with severe proteinuria and high inflammatory response.

The prevalence of anemia in young patients with CKD is likely to be relatively low. However, our results showed that the prevalences of anemia were higher in young patients (aged 18–44 years) than in patients aged 45–64 years in the G3a, G3b, and G4 categories. This result may be due to differences in characteristics between young patients with CKD in university hospitals and the general population of patients with CKD. Young patients with CKD who are regularly examined in university hospitals may represent a unique population with underlying disease. In addition, only 1173 patients in this study were aged 18–44 years (3.3% of all participants). These results suggest caution is needed when extrapolating the J-CKD-DB findings to the general population of patients with CKD.

The present study also demonstrated that the utilization rate of ESAs in Japanese patients with CKD was relatively low, compared with that of the cohort from the United States [22]. Japanese CPGs recommend starting ESA treatment after multiple confirmations of hemoglobin level ≤11.0 g/dl [31, 32]. In our dataset, the single examination of hemoglobin level may have contributed to the finding of a lower ESA utilization rate. However, the rates of hemoglobin levels within optimal ranges were low, especially in older patients; this result suggested that real-world clinical practice does not completely follow the recommendations of the CPGs. Moreover, the mean hemoglobin level was lower in patients undergoing treatment with ESAs than in patients without ESAs. These results suggested that the real-world clinical practice might consist of initiating treatment with ESAs after confirmation of a hemoglobin level much lower than 11.0 g/dl. To determine the cause of the low ESA utilization rate, the nature of the dataset should be considered. Because our dataset was not connected to the health insurance claim database, we could not determine whether patients without ESAs in our study had been treated with ESAs in other clinics or hospitals.

Several limitations of our study should be noted, in relation to its cross-sectional design. First, it is well-known that eGFR values with a single measurement of serum creatinine are prone to misclassification, especially in patients with CKD G3a who do not exhibit proteinuria, and therefore do not prove chronicity. In addition, omitting inpatients might have been a suboptimal strategy, as it may have affected the diagnosis and prevalence of CKD. Second, we were unable to obtain information regarding ferrokinetics, administration or types and dosages of iron drugs, types and dosages of ESAs, and differences in practice patterns between nephrologists and other clinicians because we could not readily convert some of the local codes regarding medical care and tests to standardized codes, despite the availability of reference mapping tables. Third, we were unable to obtain information regarding the cause of CKD, body mass index, presence or absence of diabetes and cardiovascular diseases, or blood pressure levels because these elements are not included in the SS-MIX2 system; thus, it was difficult to investigate research questions related to these variables.

In conclusion, we demonstrated the prevalence of anemia according to different international criteria for renal anemia, as well as the utilization rate of ESAs, in Japanese patients with CKD by using automatically extracted data from a nationwide, large-scale cohort study in Japan. We also assessed the influences of G category and other factors on the prevalence of anemia. Further prospective investigations including iron status and health insurance claim data, as well as analyses regarding quality indicators for CPGs, may contribute to improvements in the quality of care for patients with CKD.

## Supporting information

S1 Table Participant stratification according to eGFR category, age, and sex strata. (PDF)

S2 Table Mean hemoglobin levels according to G category, age, and sex strata. (PDF)

S3 Table Mean hemoglobin levels according to A category strata. (PDF)

S4 Table Mean hemoglobin levels in all patients, patients without severe proteinuria and without high inflammatory levels. (PDF)

S5 Table Rates of ESA non-use among patients with hemoglobin ≤11.0 g/dl according to chronic kidney disease G category and age strata. (PDF)
