# Supplementary material for: Prevalence of anemia in patients with chronic kidney disease in Japan: A nationwide, cross-sectional cohort study using data from the Japan Chronic Kidney Disease Database (J-CKD-DB)
Source: PLoS One. 2020 Jul 20;15(7):e0236132. doi: 10.1371/journal.pone.0236132 (PMC7371174; doi:10.1371/journal.pone.0236132)
Supplement: S1 Table — (PDF) [file pone.0236132.s001.pdf]

S1 Table. Participant Stratification According to eGFR Category, Age, and Sex Strata.

	eGFR category				
	G3a	G3b	G4	G5	Subtotal
eGFR range, ml/min/1.73 m^2	45–59	30–44	15–29	<15	<60
Patients	23,333	8,357	2,710	1,108	35,508
Prevalence	65.7%	23.5%	7.6%	3.1%	
Age					
18–44 years	718 (61.2%)	223 (19.0%)	131 (11.2%)	101 (8.6%)	1,173
45–64 years	5,815 (73.0%)	1,330 (16.7%)	489 (6.1%)	332 (4.2%)	7,966
65–74 years	8,146 (70.1%)	2,472 (21.3%)	688 (5.9%)	322 (2.8%)	11,628
75–84 years	6,994 (61.8%)	3,131 (27.7%)	908 (8.0%)	276 (2.4%)	11,309
≥85 years	1,660 (48.4%)	1,201 (35.0%)	494 (14.4%)	77 (2.2%)	3,432
Sex					
Men	12,661 (65.4%)	4,614 (23.8%)	1,434 (7.4%)	651 (3.4%)	19,360
Women	10,672 (66.1%)	3,743 (23.2%)	1,276 (7.9%)	457 (2.8%)	16,148
Dipstick proteinuria					
(-)	6,652 (71.1%)	2,133 (22.8%)	533 (5.7%)	39 (0.4%)	9,357
(±)	1,302 (56.7%)	693 (30.2%)	257 (11.2%)	43 (1.9%)	2,295
(1+)	778 (42.1%)	592 (32.0%)	358 (19.4%)	121 (6.5%)	1,849
(2+)	409 (32.0%)	401 (31.4%)	309 (24.2%)	158 (12.4%)	1,277
(3+)	121 (20.2%)	164 (27.4%)	189 (31.6%)	124 (20.7%)	598
(4+)	14 (21.2%)	25 (37.9%)	18 (27.3%)	9 (13.6%)	66
Not Examined	14,057 (60.2%)*	4,349 (52.0%)*	1,046 (38.6%)*	614 (55.4%)*	20,066 (56.5%)

*Prevalence of each eGFR category

Abbreviations: eGFR, estimated glomerular filtration rate
