# Supplementary material for: Prevalence of anemia in patients with chronic kidney disease in Japan: A nationwide, cross-sectional cohort study using data from the Japan Chronic Kidney Disease Database (J-CKD-DB)
Source: PLoS One. 2020 Jul 20;15(7):e0236132. doi: 10.1371/journal.pone.0236132 (PMC7371174; doi:10.1371/journal.pone.0236132)
Supplement: S2 Table — (PDF) [file pone.0236132.s002.pdf]

S2 Table. Mean Hemoglobin Levels According to G Category, Age, and Sex Strata.

Total		18–44 Y	45–64 Y	65–74 Y	75–84 Y	≥85 Y	Subtotal
G3a	Mean	13.72	13.89	13.60†	13.06†	12.42†	13.44
	SD	2.07	1.68	1.56	1.59	1.58	1.67
G3b	Mean	12.70*†	13.22*	12.95*†	12.43*†	11.88*†	12.64*
	SD	2.51	2.06	1.83	1.82	1.75	1.93
G4	Mean	11.98*	12.08*	11.90*	11.36*†	10.86*†	11.57*
	SD	2.09	2.09	2.00	1.73	1.68	1.93
G5	Mean	11.32*	11.21*	10.89*	10.62*†	9.95*†	10.90*
	SD	1.73	1.85	1.62	1.63	1.64	1.74
Overall	Mean	13.12†	13.54	13.28†	12.69†	11.94†	13.02*
	SD	2.29	1.92	1.76	1.77	1.77	1.88
Men		18–44 Y	45–64 Y	65–74 Y	75–84 Y	≥85 Y	Subtotal
G3a	Mean	14.85†	14.59	14.11†	13.55†	12.80†	14.01
	SD	1.73	1.59	1.55	1.62	1.62	1.67
G3b	Mean	13.70*	13.85*	13.47*†	12.87*†	12.17*†	13.16*
	SD	2.80	2.04	1.83	1.93	1.78	1.99
G4	Mean	12.52*	12.49*	12.40*	11.71*†	11.14*†	11.99*
	SD	2.07	2.25	2.06	1.84	1.74	2.04
G5	Mean	11.53*	11.35*	11.01*	10.64*†	10.25*†	11.03*
	SD	1.70	1.90	1.65	1.68	1.68	1.78
Overall (men)	Mean	14.04	14.17	13.77†	13.13†	12.27†	13.55
	SD	2.31	1.93	1.78	1.86	1.82	1.94
Women		18–44 Y	45–64 Y	65–74 Y	75–84 Y	≥85 Y	Subtotal
G3a	Mean	12.58†‡	13.05‡	12.93†‡	12.49†‡	12.06†‡	12.74‡
	SD	1.72	1.38	1.29	1.35	1.46	1.40
G3b	Mean	11.81*†‡	12.39*‡	12.18*†‡	11.87*†‡	11.60*†‡	12.00*‡
	SD	1.84	1.78	1.53	1.50	1.67	1.62
G4	Mean	11.46*‡	11.56*‡	11.23*‡	10.98*†‡	10.59*†‡	11.09*‡
	SD	2.00	1.76	1.70	1.50	1.57	1.67
G5	Mean	11.01*	11.01*	10.70*	10.57*	9.64*†	10.71*‡
	SD	1.74	1.77	1.55	1.55	1.54	1.67
Overall (women)	Mean	12.19†‡	12.76‡	12.61†‡	12.15†‡	11.64†‡	12.38‡
	SD	1.85	1.59	1.49	1.50	1.66	1.59

*:p<0.05 vs. G3a, †:p<0.05 vs. 45–64 Y, ‡:P<0.05 vs. Men

Mean hemoglobin levels (g/dl) are expressed as mean and SD, and were analyzed by one-way analysis of variance.

Abbreviations: SD, standard deviation
