# Supplementary material for: Prevalence of anemia in patients with chronic kidney disease in Japan: A nationwide, cross-sectional cohort study using data from the Japan Chronic Kidney Disease Database (J-CKD-DB)
Source: PLoS One. 2020 Jul 20;15(7):e0236132. doi: 10.1371/journal.pone.0236132 (PMC7371174; doi:10.1371/journal.pone.0236132)
Supplement: S3 Table — (PDF) [file pone.0236132.s003.pdf]

S3 Table. Mean Hemoglobin Levels According to A Category Strata.

Total		A1	A2	A3	Subtotal
G3a	Mean	13.51	13.54	13.69†	13.37
	SD	1.55	1.83	1.90	1.69
G3b	Mean	12.75*	12.77*	12.93*†	12.49
	SD	1.73	1.92	1.97	1.99
G4	Mean	11.48*	11.51*	11.85*†	11.40
	SD	1.60	1.76	1.98	2.06
G5	Mean	10.15*	10.39*	10.61*	11.19
	SD	1.39	1.65	1.59	1.81
Overall	Mean	13.20	13.00†	12.68†	13.01
	SD	1.70	2.00	2.16	1.89

*:p<0.05 vs. G3a, †:p<0.05 vs. A1

Mean hemoglobin levels (g/dl) are expressed as mean and SD, and were analyzed by one-way analysis of variance.

Abbreviations: SD, standard deviation
