# Supplementary material for: Prevalence of anemia in patients with chronic kidney disease in Japan: A nationwide, cross-sectional cohort study using data from the Japan Chronic Kidney Disease Database (J-CKD-DB)
Source: PLoS One. 2020 Jul 20;15(7):e0236132. doi: 10.1371/journal.pone.0236132 (PMC7371174; doi:10.1371/journal.pone.0236132)
Supplement: S4 Table — (PDF) [file pone.0236132.s004.pdf]

S4 Table. Mean Hemoglobin Levels in All Patients, Patients without Severe Proteinuria and without High Inflammatory Levels.

		All patients	Patients without UTP ≥ 3+ and Alb < 3.0 g/dl	Patients without CRP ≥ 5.0 mg/dl
G3a	Mean	13.44	13.45	13.44
	n	20335	20082	20322
	SD	1.67	1.66	1.67
G3b	Mean	12.64	12.68	12.65
	n	7318	7128	7302
	SD	1.93	1.89	1.92
G4	Mean	11.57	11.63	11.58
	n	2412	2274	2387
	SD	1.93	1.89	1.92
G5	Mean	10.90	11.01	10.92
	n	1017	944	994
	SD	1.74	1.65	1.75

Data are expressed as % of each population.

*:p<0.05 vs. total

Abbreviations: UTP, dipstick proteinuria; Alb, serum albumin; CRP, serum C-reactive protein
