# Supplementary material for: Prevalence of anemia in patients with chronic kidney disease in Japan: A nationwide, cross-sectional cohort study using data from the Japan Chronic Kidney Disease Database (J-CKD-DB)
Source: PLoS One. 2020 Jul 20;15(7):e0236132. doi: 10.1371/journal.pone.0236132 (PMC7371174; doi:10.1371/journal.pone.0236132)
Supplement: S5 Table — (PDF) [file pone.0236132.s005.pdf]

S5 Table. Rates of ESA Non-use among Patients with Hemoglobin ≤11.0 g/dl According to Chronic Kidney Disease G Category and Age Strata.

		18–44 Y	45–64 Y	65–74 Y	75–84 Y	≥85 Y	Subtotal
G4	%	83.8	81.0	85.9	83.0	87.7	84.5
G5	%	54.8*	67.7*	62.4*	62.9*	77.8	64.9*
G4+G5	%	68.4	73.9	75.8	77.3	85.8†	77.5

Data are expressed as % of each population.

*:p<0.05 vs. G4, †:p<0.05 vs. 45–64 Y

Abbreviations: Y, years; Hb, hemoglobin; ESA, erythropoiesis-stimulating agent
